# A candidate PPARγ to GPX4 axis in ferroptosis suppression and platinum resistance in bladder cancer: evidence appraisal and a testable research agenda

**DOI:** 10.7150/jca.139186

**Published:** 2026-09-24

**Authors:** Shenjuan Li, Hongwei Peng, Gang Li, Ziyu Wan, Mengxue Yu, Sheng Tu, Fangjin Chen, Lingao Ju, Gang Wang, Kaiyu Qian, Yu Xiao, Zilin Xu

**Affiliations:** 1 Department of Biological Repositories, Zhongnan Hospital of Wuhan University, Wuhan, 430071, China.; 2 Human Genetic Resources Preservation Center, Zhongnan Hospital of Wuhan University, Wuhan, 430071, China.; 3 Brain Glioma Center & Department of Neurosurgery, Zhongnan Hospital of Wuhan University, Wuhan, 430071, China.; 4 Department of Urology, Hubei Key Laboratory of Urological Diseases, Zhongnan Hospital of Wuhan University, Wuhan, 430071, China.; 5 Center for Quantitative Biology, School of Life Sciences, Peking University, Beijing, 100871, China.; 6 Department of Laboratory Medicine, Zhongnan Hospital of Wuhan University, Wuhan, 430071, China.; 7 Laboratory of Precision Medicine, Zhongnan Hospital of Wuhan University, Wuhan, 430071, China.; 8 Wuhan Research Center for Infectious Diseases and Cancer, Chinese Academy of Medical Sciences, Wuhan, 430071, China.

**Keywords:** bladder cancer, ferroptosis, GPX4, PPARγ, platinum resistance, evidence appraisal, lipid peroxidation

## Abstract

Cytotoxic chemotherapy no longer holds an uncontested place in the treatment of muscle-invasive bladder cancer, although platinum-based therapy remains relevant in selected perioperative, bladder-preservation, upper-tract, and salvage settings. Mechanisms of platinum resistance therefore retain clinical importance. Ferroptosis, an iron-dependent form of regulated cell death constrained by glutathione peroxidase 4 (GPX4), has been implicated in treatment tolerance and drug-tolerant persister states. Here, we provide a narrative evidence appraisal of peroxisome proliferator-activated receptor gamma (PPARγ) as a candidate upstream regulator of GPX4-dependent ferroptosis resistance in bladder cancer. Two mechanistic arms are considered. The first proposes transcriptional regulation of GPX4 by PPARγ; however, positive regulation in bladder cancer is supported only by expression co-variation and in silico motif prediction, while evidence from another cellular context indicates that the direction of PPARγ-GPX4 regulation can be reversed. The second proposes that reduced SIRT1 activity could alter GPX4 acetylation and thereby influence its post-translational regulation. Although GPX4 acetylation has been directly demonstrated in non-urothelial systems, GPX4 has not been established as a SIRT1 substrate, and SIRT1-dependent control of GPX4 ubiquitination or turnover remains untested. We therefore present the PPARγ-GPX4 relationship as a context-dependent, testable hypothesis rather than an established “dual-lock” mechanism, and outline the experiments required to establish transcriptional directionality, post-translational regulation, ferroptosis dependence, and platinum sensitization in urothelial models.

## 1. Introduction

The systemic treatment of bladder cancer (BLCA) has changed faster than the resistance literature that accompanies it. In cisplatin-ineligible or cisplatin-declining muscle-invasive bladder cancer (MIBC), the phase III KEYNOTE-905/EV-303 trial established perioperative enfortumab vedotin plus pembrolizumab as an effective cystectomy-based treatment strategy 1. Subsequently, in cisplatin-eligible MIBC, the phase III KEYNOTE-B15/EV-304 trial reported superior event-free survival, overall survival and pathological complete response with perioperative enfortumab vedotin plus pembrolizumab compared with neoadjuvant cisplatin plus gemcitabine 2. On the basis of these data, the U.S. FDA expanded the perioperative indication in July 2026 to all patients with MIBC who are candidates for cystectomy. Importantly, platinum-containing perioperative therapy also remains clinically relevant in cisplatin-eligible MIBC: the phase III NIAGARA trial demonstrated improved event-free and overall survival with perioperative durvalumab combined with neoadjuvant gemcitabine plus cisplatin compared with neoadjuvant chemotherapy alone 3. In advanced disease, enfortumab vedotin plus pembrolizumab is the preferred first-line standard for patients with advanced urothelial carcinoma who are fit for combination therapy, irrespective of cisplatin eligibility, and biomarker-defined later-line options include erdafitinib for susceptible FGFR3-altered disease and trastuzumab deruxtecan for previously treated HER2 IHC 3+ disease 4-6. Claims that cytotoxic chemotherapy is indispensable to the management of MIBC can no longer be sustained, and we do not make them.

Platinum exposure has nonetheless not disappeared from practice, and this is the premise on which the present review rests. Platinum-based adjuvant chemotherapy remains the standard after radical nephroureterectomy for eligible patients with locally advanced upper tract urothelial carcinoma 3, 7. Platinum remains the cytotoxic backbone of bladder preservation strategies, including protocols in which a stringently defined clinical complete response after cisplatin-based chemotherapy plus a checkpoint inhibitor permits cystectomy to be deferred 8. Platinum is used in salvage lines after progression on antibody-drug conjugate (ADC) or checkpoint inhibitor therapy, a setting in which options remain limited. Conventional MVAC is now rarely used in contemporary practice, whereas dose-dense MVAC remains a guideline-recognized cisplatin-based option in selected patients, with substantial regional variation in uptake. Two facts follow. Resistance to platinum continues to determine outcomes for a substantial number of patients, and the mechanisms responsible remain incompletely defined, with candidate explanations spanning nucleoside transport, drug efflux and DNA damage response signaling, none of which has produced a validated means of restoring sensitivity.

This review concerns urothelial carcinoma of the bladder and does not extend to upper tract or urethral tumors, for a reason that we state rather than assume. The evidence base for the PPARγ axis is bladder specific: PPARG genomic activation, the luminal transcriptional programs in which it operates, and the survival associations discussed below derive from cohorts in which upper tract and urethral tumors are excluded or too few to analyze separately. Upper tract urothelial carcinoma differs in FGFR3 alteration frequency, in the prevalence of mismatch repair deficiency and Lynch syndrome, and in immune contexture, so applying the model there would be extrapolation rather than synthesis 9, 10. This restriction is analytical and not a statement of clinical priority. Because platinum-based adjuvant chemotherapy remains standard in upper tract disease, the clinical stake in platinum resistance is arguably higher there than in the bladder, and we return to this in the research agenda.

Peroxisome proliferator-activated receptor gamma (PPARγ), a ligand-activated nuclear receptor that coordinates transcriptional programs that govern lipid metabolism, adipogenesis, and cellular redox balance 11, is aberrantly activated in a substantial subset of urothelial carcinomas and has been linked, in a context-dependent manner, to luminal lineage programs, tumor cell proliferation, and immune exclusion 12-16. Transcriptomic analyses of BLCA patient cohorts revealed a significant inverse correlation between PPARγ pathway activity and ferroptosis gene-expression scores 14, 17, suggesting that PPARγ is a candidate upstream regulator of ferroptosis suppression in this disease.

This mini-review synthesizes mechanistic, transcriptomic, and clinical evidence to outline a working model in which PPARγ may suppress ferroptosis in BLCA via a two-arm mechanism termed the “dual-lock” hypothesis. This term is employed as a label for the model under examination rather than as a description of an established circuit.

## 2. Methods

### 2.1 Design

This is a narrative mechanistic synthesis of published literature. It is not a systematic review and does not claim compliance with PRISMA. No new patient data were collected, and no computational analysis, statistical modeling or code was generated by the authors. Every cohort level or quantitative statement in this article is attributed to the primary publication that reported it.

### 2.2 Search strategy

We searched PubMed, Web of Science Core Collection and Embase from inception to 8 September 2026, using combinations of the following terms: (“bladder cancer” OR “urothelial carcinoma”) AND (“ferroptosis” OR “GPX4” OR “lipid peroxidation”); (“PPARG” OR “PPARgamma” OR “peroxisome proliferator-activated receptor gamma”) AND (“bladder” OR “urothelial”); (“PPARG” OR “PPARgamma”) AND (“GPX4” OR “ferroptosis”); (“SIRT1” OR “SIRT3” OR “sirtuin”) AND (“GPX4” OR “acetylation” OR “deacetylation” OR “ubiquitination”); (“GPX4”) AND (“protein stability” OR “ubiquitin” OR “ubiquitination” OR “acetylation” OR “deacetylation”). Clinical practice evidence was identified through a separate search of (“muscle-invasive bladder cancer”) AND (“neoadjuvant” OR “perioperative” OR “first-line”) restricted to randomized phase III trials, guideline documents and regulatory decisions issued from January 2023 onward, supplemented by hand searching of the reference lists of retrieved trials.

### 2.3 Eligibility

We included English-language primary research articles, randomized trials, guideline statements, and reviews addressing the ferroptosis machinery, PPARγ biology in urothelial tissue, sirtuin substrate biology, or the systemic treatment of urothelial carcinoma. Reviews were used for contextual background and reference tracing but were not used as primary evidentiary support for claims central to the proposed model. We excluded conference abstracts without subsequent full publication and preprints. For mechanistic claims central to the proposed model, we preferentially relied on the primary experimental report rather than a secondary citation.

### 2.4 Evidence appraisal

Because the mechanistic literature bearing on this axis is uneven, we graded every component claim of the model against three levels rather than presenting them as a continuous narrative. Level A denotes a claim supported by direct experimental evidence in urothelial cells or tissue. Level B denotes a claim supported by direct experimental evidence in another tissue or cell type, requiring extrapolation to urothelium. Level C denotes a claim supported only by correlation, in silico prediction, or analogy, without direct experimental evidence supporting the specific proposed relationship. Direct evidence from another cellular context that contradicts the proposed direction of effect is reported separately rather than taken as support for the claim. The grading is reported in Table 1, together with the specific experiment that would move each claim to a higher level.

### 2.5 Limitations of the method

A narrative synthesis constructed around a proposed model is susceptible to selection bias favoring that model. We have attempted to constrain this by grading claims explicitly, by stating for each arm what evidence is absent, and by specifying in Section 5 the observations that would refute the model rather than only those that would support it. Readers should nonetheless treat the coherence of the model as a property of its construction and not as evidence for its truth.

## 3. Ferroptosis as a determinant of chemosensitivity in BLCA

### 3.1 Core ferroptotic machinery

Ferroptosis is initiated by the iron-catalyzed peroxidation of polyunsaturated fatty acid-containing phospholipids, generating toxic lipid hydroperoxides that disrupt membrane integrity and trigger cell death 18, 19. The central checkpoint is glutathione peroxidase 4 (GPX4), which catalytically neutralizes phospholipid hydroperoxides using reduced glutathione as a co-substrate, thereby preventing oxidative membrane rupture 20, 21. Key regulatory nodes include: the cystine/glutamate antiporter system Xc^-^, which supplies cysteine for glutathione biosynthesis and is pharmacologically inhibited by erastin; acyl-CoA synthetase long-chain family member 4 (ACSL4), which channels arachidonate and other polyunsaturated fatty acids into pro-ferroptotic phospholipid pools 22; and the labile intracellular iron pool, which amplifies lipid radical chain reactions through Fenton chemistry 23. Importantly, the CoQ oxidoreductase FSP1 has emerged as a GPX4-independent ferroptosis suppressor, indicating that some tumors can rely on more than one anti-ferroptotic safeguard 24. Even so, in many solid-tumor models GPX4 still appears to be the key node, given the reproducible ferroptotic death observed after pharmacologic GPX4 inhibition or genetic GPX4 loss 20.

### 3.2 Ferroptosis suppression and resistance in BLCA

In BLCA, ferroptosis-associated programs are often lower in tumor cells than in normal urothelium. Single-cell studies that integrated EpiTrace-based chromatin accessibility and evolutionary tracing suggest that stem-like, epigenetically plastic BLCA states show weaker ferroptosis-related gene signatures than more differentiated tumor populations 17, 25. This includes a TM4SF1-positive subpopulation associated with intratumoral heterogeneity and adaptive epigenetic plasticity 17, 25. This finding is consistent with previous work showing that plastic cell states can evade ferroptosis and persist under therapeutic pressure 26.

The mechanistic basis for this idea comes in part from two articles by Viswanathan *et al*. and Hangauer *et al*. These studies showed that drug-tolerant persister cells in several tumor types rely on GPX4 to remove lipid hydroperoxides, making them selectively vulnerable to GPX4 inhibition 26, 27. Although this mechanism has not been fully established in urothelial cancer, it raises the possibility that GPX4-mediated suppression of ferroptosis contributes to chemotherapy tolerance in BLCA. In line with this possibility, several preclinical studies have reported that ferroptosis induction can improve the efficacy of conventional chemotherapies 23. These findings point to a more specific question in BLCA: which upstream pathways preserve GPX4 expression or protein stability during cytotoxic stress?

## 4. PPARγ in BLCA: a context-dependent regulator of ferroptosis

PPARγ is a ligand-activated transcription factor that forms a heterodimer with RXR and binds PPAR response elements to regulate gene expression in a metabolism-dependent manner 11. Although it is best known for its roles in adipogenesis, lipid storage, and insulin sensitivity, PPARγ signaling can also be co-opted in epithelial cancers, where it influences lipid metabolism, oxidative-stress responses, and inflammatory signaling. Its effects, however, vary substantially across tumor types. In some settings, such as early-stage colorectal carcinoma, PPARγ activation has been associated with differentiation and growth restraint 11. These context-dependent effects suggest that PPARγ does not function as a uniformly pro- or anti-tumorigenic factor, but instead acts according to cell state and local metabolic constraints.

In BLCA, genomic, transcriptomic, and protein-level studies support aberrant activation of PPARγ signaling in a substantial subset of tumors, particularly those with luminal features 12-16. Integrated transcriptomic analysis of BLCA cohorts using the EpiTrace platform further suggests an inverse association between PPARγ pathway activity and ferroptosis-related transcriptional programs 17, 28. However, correlations involving individual components such as GPX4 and SIRT1 should be interpreted as hypothesis-generating rather than evidence of direct regulation. Patients classified as “low-ferroptosis” by a ferroptosis gene-expression score (an expression state enriched for higher PPARγ pathway activity) showed worse overall survival 17, 28. This survival pattern may also reflect differences in molecular subtype distribution, tumor purity and immune content, or heterogeneity in treatment exposure and timing, and therefore should be interpreted cautiously. Together, these clinical genomic observations motivate further studies to clarify whether PPARγ contributes to ferroptosis suppression in BLCA and whether it could be evaluated as a biomarker linked to adverse outcomes.

## 5. PPARγ “dual-lock” mechanism for GPX4 maintenance

As illustrated in Figure 1 (middle panel), the available evidence is consistent with a working model in which PPARγ may maintain GPX4 at anti-ferroptotic levels through two mechanistically distinct regulatory arms that together form a putative “dual-lock” circuit.

### 5.1 First arm: context-dependent transcriptional regulation of GPX4 by PPARγ

The first proposed arm concerns transcriptional regulation of GPX4 by PPARγ. The rationale for a positive regulatory relationship in BLCA is that PPARγ heterodimerizes with RXR, binds canonical response elements, and regulates antioxidant and lipid-metabolizing gene networks 11, while GPX4 expression has been reported to co-vary with PPARγ pathway activity in BLCA transcriptomic cohorts 14, 17. Consistent with this possibility, the GPX4 proximal promoter contains a sequence resembling a PPAR response element. However, the presence of this motif alone does not show that PPARγ binds the GPX4 promoter in urothelial cells, nor does it indicate whether PPARγ would activate or repress GPX4 transcription.

This point is illustrated by recent work in *Mycobacterium tuberculosis*-infected murine macrophages. In that setting, Pu *et al*. found increased PPARγ occupancy at the Gpx4 promoter by ChIP-seq and ChIP-qPCR, but this was accompanied by reduced, rather than increased, Gpx4 expression 29. The authors also observed lower H3K27ac levels and decreased chromatin accessibility at the locus. Mechanistically, PPARγ recruited the NCOR/SMRT corepressor complex, and depletion of either corepressor restored Gpx4 expression. Rosiglitazone treatment further strengthened GPX4 suppression 29. Thus, PPARγ binding at the Gpx4 locus does not necessarily imply transcriptional activation, and its effect appears to depend on the chromatin environment and available coregulators.

The strength and direction of the bladder-cancer evidence must therefore be stated accurately. Expression co-variation across tumors is compatible with direct transcriptional control but is equally compatible with indirect regulation through intermediate transcription factors, shared dependence on luminal differentiation state, or subtype-related confounding 14, 17. No study has established that PPARγ positively regulates GPX4 transcription in urothelial cells, and no bladder-cancer study has directly tested GPX4 transcription after genetic or pharmacological PPARγ perturbation. Accordingly, a positive PPARγ-GPX4 transcriptional relationship in BLCA remains a Level C hypothesis.

If this arm were confirmed in BLCA, its biological interest would lie in establishing a context-specific mode of GPX4 regulation that may operate alongside the Nrf2-antioxidant response element pathway implicated in ferroptosis resistance elsewhere 30 and other previously described mechanisms controlling GPX4 expression 21. If it were refuted, the observed co-variation between PPARγ activity and GPX4 expression 14, 17 would still require explanation, potentially through luminal differentiation programs or other shared upstream regulators. Future experiments must therefore determine not only whether PPARγ occupies the GPX4 locus, but also whether such occupancy results in transcriptional activation, repression, or no functional effect in urothelial cells.

### 5.2 Second arm: candidate post-translational regulation of GPX4 through the PPARγ-SIRT1 axis

The second arm is proposed to operate at the post-translational level through SIRT1, an NAD^+^-dependent class III protein deacetylase that regulates diverse substrates that govern cellular metabolism, stress response, and protein stability 31, 32. PPARγ and SIRT1 engage in reciprocal regulatory crosstalk. SIRT1 deacetylates and represses PPARγ transcriptional activity in metabolic tissues 33, whereas PPARγ can suppress SIRT1 expression or activity in other contexts, including BLCA-associated signaling models 34, 35. SIRT1 is well known as a deacetylase capable of modifying multiple non-histone protein substrates critical to oncogenic signaling and cell death regulation 31, 32. More broadly, acetylation and ubiquitination can compete for shared lysine residues in certain proteins, such that deacetylation may facilitate ubiquitination and proteasomal turnover in a substrate-dependent manner 36. GPX4 protein stability is independently subject to ubiquitin-dependent regulation 37, 38. Importantly, GPX4 acetylation itself has now been demonstrated experimentally in non-urothelial systems. K90 acetylation has been shown to preserve GPX4 enzymatic activity and suppress ferroptosis 39, while studies of mitochondrial GPX4 have linked SIRT3-dependent deacetylation to regulation of GPX4 acetylation, abundance, localization, and ferroptosis 40, 41. These findings establish GPX4 acetylation as a biologically relevant post-translational modification but also indicate that its functional consequences are context- and site-dependent. They do not establish SIRT1 as a GPX4 deacetylase or demonstrate that GPX4 acetylation prevents ubiquitin-dependent degradation.

The evidentiary position of the specific SIRT1-dependent arm nevertheless remains weak. Although GPX4 acetylation has been directly demonstrated and functionally linked to ferroptosis in non-urothelial systems 39-41, GPX4 has not been established as a SIRT1 substrate. No study has demonstrated direct SIRT1-dependent deacetylation of GPX4 in urothelial cells or, to our knowledge, in another cellular context. Moreover, the available GPX4 acetylation studies do not establish the specific relationship proposed here, namely that SIRT1-dependent deacetylation facilitates GPX4 ubiquitination and proteasomal turnover. General competition between acetylation and ubiquitination has been demonstrated for other substrates 36, and GPX4 protein stability is independently regulated by ubiquitination 37, 38, but a causal acetylation-ubiquitination switch controlling GPX4 degradation has not been demonstrated. Accordingly, the proposed PPARγ-SIRT1-GPX4 stabilization mechanism remains an inference that we grade as Level C in Table 1.

Two alternative explanations must be excluded before this arm could be accepted. First, any observed increase in GPX4 protein under high PPARγ conditions may follow entirely from the transcriptional arm, in which case no post-translational mechanism is required. Second, SIRT1 loss has broad metabolic consequences, including altered NAD^+^ flux and changes in mitochondrial function, that could affect ferroptosis sensitivity without acting on GPX4 at all. Distinguishing these possibilities requires direct assessment of SIRT1-GPX4 interaction, site-specific GPX4 acetylation, GPX4 ubiquitination and protein half-life after SIRT1 gain- and loss-of-function, rather than measurement of steady-state GPX4 abundance alone.

### 5.3 Established alternative PPARγ-dependent ferroptosis-suppressive pathways in BLCA

GPX4 is unlikely to represent the only ferroptosis effector downstream of PPARγ in BLCA, and current direct evidence also supports alternative effectors. EMP1 loss has been reported to enhance PPARγ activity and increase SLC7A11 expression 42, whereas a recent study identified PLA2G2F as a direct transcriptional target and major functional mediator of PPARγ-dependent ferroptosis resistance in BLCA cells 43. Notably, PPARγ agonism produced only modest, if any, regulation of GPX4 in the latter study 43. These findings position the proposed PPARγ-GPX4 relationship as one candidate branch within a broader PPARγ-centered anti-ferroptotic network rather than as the established dominant effector pathway.

## 6. Translational implications and research priorities

### 6.1 What the model predicts, and what would refute it

In malignant urothelial cells, the proposed model predicts that PPARγ antagonism should reduce GPX4 abundance, increase lipid peroxidation, and enhance platinum sensitivity. If PPARγ maintains GPX4 through the proposed mechanisms, this effect should be reversible by ferrostatin-1 or liproxstatin-1 and by GPX4 overexpression. If PPARγ antagonism reduces viability without a lipid-peroxidation signature, or if the effect is not rescued by ferroptosis inhibitors, the ferroptosis interpretation would fail even if an antitumor effect were observed. This distinction has not been addressed in the existing PPARγ-targeting literature in BLCA, in which proliferation, cell-cycle progression, survival, and motility rather than ferroptosis were the principal endpoints 14, 15, 44. More recent work has demonstrated that PPARγ inhibition can sensitize BLCA cells to ferroptosis through a PLA2G2F-dependent mechanism 43; however, that study does not establish a PPARγ-GPX4 mechanism or demonstrate GPX4-dependent platinum sensitization. Thus, the specific prediction proposed here remains unresolved.

### 6.2 Pharmacological feasibility and scheduling

The pharmacological position must be described without optimism. No PPARγ antagonist is approved for clinical use. GW9662 is an irreversible covalent antagonist developed as a laboratory tool, with no human pharmacokinetic, pharmacodynamic, or safety data 45. An alternative drug-development strategy may be to exploit mechanisms that promote PPARγ degradation, such as the SIAH1/2-dependent pathway described in luminal BLCA 44.

The direction of PPARγ pharmacology also deserves careful consideration. The clinically established PPARγ ligands are agonists, including the thiazolidinedione hypoglycemic agents pioglitazone and rosiglitazone. As discussed above, the direction of GPX4 regulation cannot be inferred from PPARγ agonist activity alone and appears to depend on cellular and coregulator context. Accordingly, concurrent thiazolidinedione exposure should not presently be assumed either to enhance or to impair platinum response through GPX4 in BLCA. Nevertheless, pharmacoepidemiological analyses of thiazolidinedione exposure in platinum-treated urothelial carcinoma could still be informative as exploratory tests of PPARγ-related treatment interaction, provided that they are interpreted independently of any prespecified GPX4-mediated direction of effect. Such analyses would require adjustment for diabetes status and severity, glycemic control, renal function, molecular subtype where available, and other treatment-related covariates. Given the historical debate over pioglitazone and BLCA risk 46, such an analysis retains independent clinical interest.

No optimal scheduling strategy can currently be specified. If a clinically tractable PPARγ antagonist becomes available, intermittent exposure around platinum administration could be compared preclinically with continuous dosing. The optimal schedule should be determined empirically from tumor-cell target engagement, GPX4 and ferroptosis-effector dynamics, myeloid-cell responses, platinum pharmacology, and systemic toxicity rather than inferred solely from the proposed GPX4 mechanism.

## 7. Discussion

The evidence synthesized here supports evaluation of a candidate model in which PPARγ-associated regulation may contribute to GPX4 maintenance and ferroptosis resistance in BLCA 11, 14, 17, 26. The proposed transcriptional and post-translational arms remain unvalidated in urothelial models and should therefore be regarded as testable components rather than an established dual-lock circuit. If validated, coupling between these two regulatory layers could help explain how BLCA cells maintain anti-ferroptotic protection during cytotoxic stress. This would be consistent with the reported dependence of therapy-resistant cancer cell states on GPX4-mediated lipid peroxide detoxification 26, 27.

### 7.1 Context dependence and unresolved questions

The proposed pro-tumor role of PPARγ in BLCA contrasts with findings from other epithelial cancers, including colorectal and prostate cancer, where PPARγ activation has been associated with differentiation, cell-cycle restraint, and growth inhibition 11. In BLCA itself, the available evidence also points to strong context dependence: PPARγ activity is most closely associated with luminal differentiation programs, genomic activation, and immune-excluded tumor states 12, 13, 16. One explanation is that PPARγ controls a distinct set of downstream transcriptional programs in urothelial tumors. This rewiring may reflect the lipid-rich luminal environment of the bladder, tumor-specific metabolic changes, and epigenetic constraints imposed by the urothelial lineage. Whether bladder-relevant endogenous ligands, including fatty-acid derivatives shaped by diet or inflammation, bias PPARγ signaling toward a therapy-resistant state remains an open but testable question.

SIRT1 introduces a related uncertainty. Across tumor types, SIRT1 has been reported to have both tumor-suppressive and tumor-promoting functions 31, 32. Therefore, if SIRT1 repression in BLCA is confirmed, it would support a model in which reduced SIRT1 activity stabilizes GPX4 and promotes cell survival in this context. It remains to be determined whether this reflects urothelium-specific differences in SIRT1 substrate usage, or a broader context-dependent role for SIRT1 within ferroptosis-associated pathways.

The macrophage data also caution against assuming that PPARγ targeting will have the same effect in every cellular compartment. In *Mycobacterium tuberculosis*-infected macrophages, PPARγ inhibition increases GPX4 expression and reduces lipid peroxidation 29. Thus, systemic modulation of PPARγ could have different ferroptotic consequences in malignant urothelial cells and in myeloid cells within the tumor microenvironment. Future work should therefore separate tumor-cell-intrinsic effects from microenvironmental effects, ideally using co-culture systems and immunocompetent models.

### 7.2 Potential confounding in clinical association studies

Clinical association studies will also require careful design. Analyses linking PPARγ activity, ferroptosis-related transcriptional programs, and patient outcomes should account for molecular subtype, tumor purity, and prior or ongoing treatment exposure. PPARγ signaling is closely linked to luminal differentiation in BLCA 12, 13, 16, whereas bulk ferroptosis-related expression scores may be influenced by stromal and immune-cell composition. In addition, retrospective cohorts frequently lack complete information on platinum exposure, treatment sequence, and response endpoints. Accordingly, subtype-stratified and purity-adjusted analyses in cohorts with documented treatment exposure and pathological or clinical response endpoints will be required before associations involving PPARγ and ferroptosis-related programs can be interpreted as evidence of platinum resistance.

### 7.3 Current research gaps

Several important limitations constrain the current evidence. First, a causal role of PPARγ-associated ferroptosis suppression in clinical chemotherapy resistance has not been established in prospective human cohorts; survival correlations derived from the TCGA and related cohorts remain retrospective and subject to confounding by molecular subtype and other clinical variables 14, 17. Second, the relative contributions of the two proposed dual-lock arms across BLCA molecular subtypes, basal/squamous versus luminal, have not been quantified, and predictive biomarkers distinguishing patients predominantly dependent on transcriptional versus post-translational GPX4 maintenance remain undefined. Third, the systemic metabolic effects of PPARγ antagonism (on adipogenesis, cardiovascular function, and immune cell polarization) may narrow the therapeutic window in patients with metabolic comorbidities, and clinically acceptable dosing regimens for GW9662 or next-generation PPARγ antagonists have not been established. Fourth, the relationship between the PPARγ-ferroptosis axis and other resistance mechanisms in BLCA, including AR-driven epigenetic reprogramming 47 and the POLD1-MYC oncogenic axis 48, has not been systematically characterized, leaving the question of whether these pathways converge or operate independently unanswered.

### 7.4 Relevance across treatment modalities

The ferroptosis suppressive mechanism discussed here is proposed for platinum agents, and its extension to other modalities requires care. Glutathione dependent detoxification and lipid peroxidase activity are directly implicated in tolerance to platinum 30, and GPX4 dependence characterizes drug tolerant persister states across tumor types 26, 27. These are the settings in which the model is plausible, and we restrict our claim to them.

Extension to ADCs does not follow automatically, for two reasons. First, initial intracellular payload delivery is target dependent. Enfortumab vedotin binds Nectin-4 on antigen-expressing cells, undergoes internalization, and releases monomethyl auristatin E (MMAE) intracellularly after linker cleavage. However, released MMAE is membrane permeable and may subsequently exert a bystander effect on neighboring antigen-low or antigen-negative cells, as demonstrated by direct comparisons of membrane-permeable and -impermeable auristatin payloads in admixed tumor models 49, 50. Thus, resistance can arise upstream of payload action through reduced antigen expression, impaired internalization, or altered intracellular processing, but the biological effects of the released payload are not necessarily restricted to the initially targeted cell. Accordingly, any interaction between ferroptosis regulation and enfortumab vedotin sensitivity would need to distinguish effects on target-dependent ADC delivery from effects on downstream MMAE cytotoxicity. Second, payload class differs across approved agents. Enfortumab vedotin carries monomethyl auristatin E, a microtubule inhibitor, whereas trastuzumab deruxtecan carries DXd, a topoisomerase I inhibitor of the camptothecin class, approved for HER2 IHC 3+ solid tumors including BLCA 6. Lipid peroxidation dependent death is not equally implicated in tolerance to microtubule inhibitors and to topoisomerase I inhibitors, and neither has been tested in relation to the axis described here. We therefore state that the relationship between GPX4 dependent ferroptosis suppression and ADC payload sensitivity in urothelial carcinoma is unknown, and we identify it as a question for investigation rather than as an argument for the relevance of our model.

### 7.5 Future directions

Future studies should first establish whether genetic or pharmacological suppression of PPARγ reproducibly enhances platinum-induced ferroptosis in molecularly defined BLCA models, and whether any such effect depends on GPX4 rather than alternative PPARγ-regulated ferroptosis pathways. Mechanistic validation should include direct assessment of GPX4 transcription, protein stability, lipid peroxidation, and ferroptosis rescue, together with patient-derived models and immunocompetent systems capable of distinguishing tumor-cell-intrinsic from myeloid-cell responses. In parallel, composite biomarker strategies integrating PPARγ activity, ferroptosis-related programs, and SIRT1 status could be explored in retrospective, treatment-annotated cohorts, while PPARγ-ferroptosis profiling could be incorporated into conditional reprogramming cell-based *ex vivo* drug-sensitivity workflows as a preclinical platform for testing individualized chemosensitization hypotheses 51. Whether the proposed PPARγ-GPX4 relationship extends to other genitourinary malignancies should also be investigated. Clinical evaluation of PPARγ antagonism should be considered only if the mechanism is validated in BLCA and a clinically tractable antagonist with acceptable pharmacokinetic and safety properties becomes available.

## Figures and Tables

**Figure 1:**
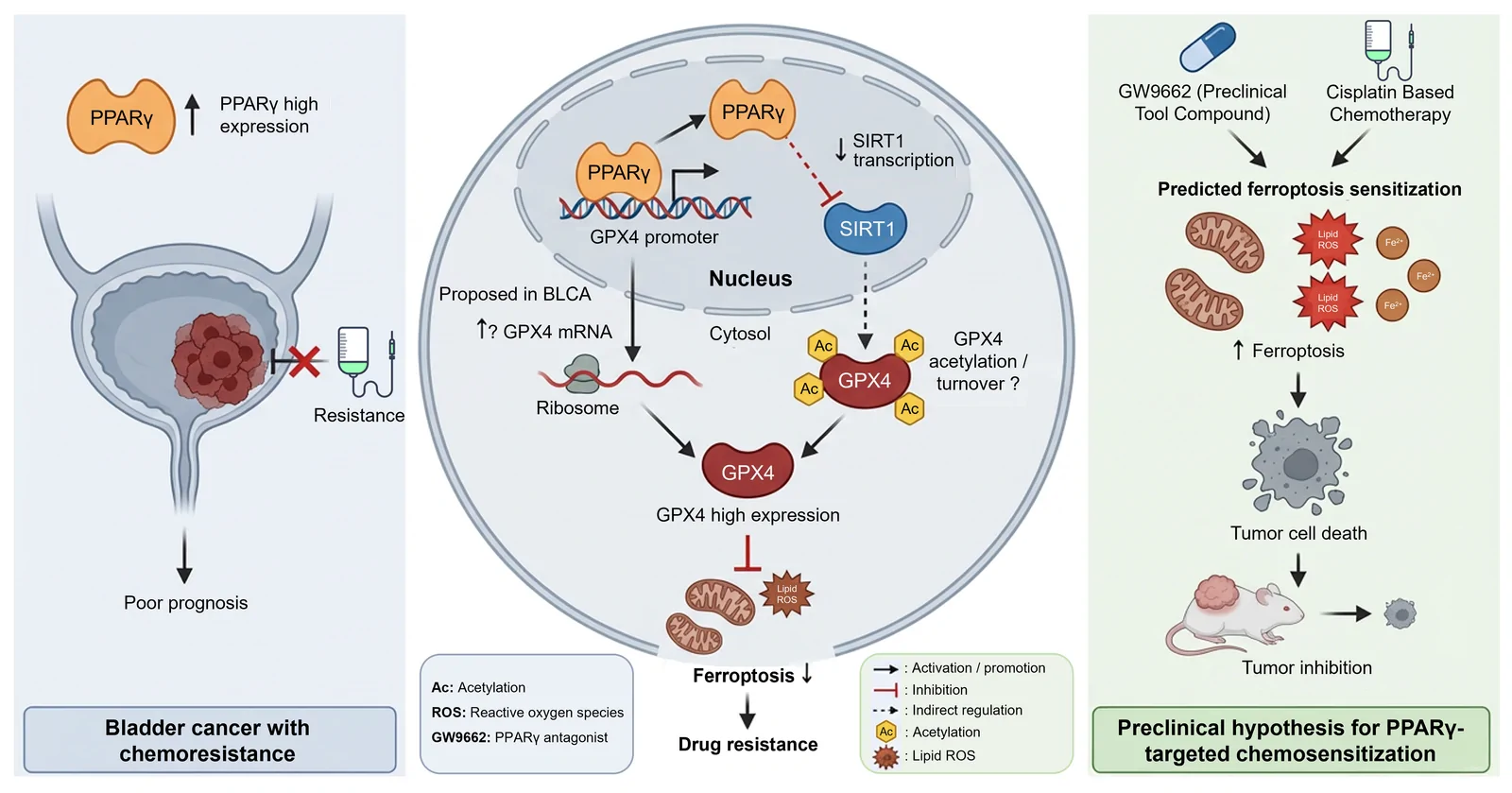
** Proposed PPARγ-associated model of GPX4 regulation and ferroptosis resistance in BLCA. (Left panel)** Aberrant PPARγ activation in BLCA has been associated with luminal lineage programs, tumor cell proliferation, and immune exclusion, supporting further investigation of the PPARγ-ferroptosis axis as a candidate therapeutic target 12-16, 44. **(Middle panel)** Proposed mechanistic basis of a PPARγ “dual-lock” circuit for GPX4 maintenance and ferroptosis suppression. In the first arm (putative, transcriptional), nuclear PPARγ is proposed to increase GPX4 transcription, potentially through interaction with a regulatory element in the GPX4 proximal promoter, which is consistent with the established DNA-binding properties of PPARγ 11. In the second arm (hypothesized, post-translational), elevated PPARγ activity is proposed to be associated with reduced SIRT1 expression or activity 34, 35. GPX4 acetylation has been directly demonstrated in non-urothelial systems and can influence GPX4 function in a context- and site-dependent manner 39-41. However, GPX4 has not been established as a SIRT1 substrate, and the proposed link between SIRT1-dependent GPX4 deacetylation, ubiquitination, and proteasomal turnover remains untested. General acetylation-ubiquitination crosstalk and independent ubiquitin-dependent regulation of GPX4 therefore provide only indirect support for this component of the model 36-38. If operative in urothelial cells, the two proposed arms could influence GPX4 abundance and/or function, thereby altering lipid-peroxide detoxification and ferroptosis sensitivity 19, 20, which may contribute to drug resistance. The arrows indicate the following: solid black, activation/promotion; flat-headed red, inhibition; dashed, indirect regulation; Ac, acetylation; starburst, lipid ROS. **(Right panel)** This panel summarizes a testable hypothesis: combining pharmacologic PPARγ antagonism (e.g., the laboratory tool compound GW9662) with intravenous platinum-based chemotherapy may increase ferroptotic susceptibility in urothelial tumor cells, as indicated by higher lipid peroxidation and changes in labile Fe^2+^/Fe^3+^ dynamics 12, 14, 23, 26, 27, 44. In turn, this combination may enhance cytotoxic responses in platinum-exposed, clinically relevant settings, pending the development of a clinically tractable PPARγ antagonist with acceptable pharmacokinetic and safety profiles.

**Table 1:** Evidence appraisal of the component claims of the proposed PPARγ to GPX4 axis in BLCA.

	Claim	Evidence available	Level	Experiment required to raise the level
1	GPX4 is a central suppressor of ferroptosis in solid tumor cells	Genetic ablation and pharmacological inhibition induce ferroptosis across multiple tumor models 20, 26, 27.	B for extrapolation to BLCA	GPX4 loss of function in urothelial lines and patient derived models with lipid peroxidation readout
2	PPARγ is genomically activated in a subset of BLCA and contributes to luminal programs	Genomic, transcriptomic and functional studies in BLCA 12, 13, 15, 16.	A	Established
3	PPARγ pathway activity is reported to be inversely associated with ferroptosis gene-expression scores in BLCA cohorts	Descriptive bulk and single-cell transcriptomic associations 14, 17.	C, association only	Subtype-stratified and purity-adjusted multivariable analysis in cohorts with documented platinum exposure and treatment-response endpoints
4	PPARγ positively regulates GPX4 transcription in BLCA	In BLCA, support is limited to expression co-variation and in silico motif prediction 14, 17. In Mycobacterium tuberculosis-infected macrophages, direct ChIP-based evidence demonstrates PPARγ occupancy at the Gpx4 locus with NCOR/SMRT-dependent transcriptional repression rather than activation 29.	C for positive regulation in BLCA; Level B counterevidence for the direction of effect from macrophages	PPARγ ChIP-seq/CUT&RUN, H3K27ac profiling, ATAC-seq, and coregulator occupancy at the GPX4 locus in urothelial models; GPX4 mRNA and protein after genetic and pharmacological PPARγ perturbation; promoter-reporter assays with motif mutagenesis and subtype-specific validation
5	PPARγ activity reduces SIRT1 expression or activity	Reciprocal PPARγ and SIRT1 regulation in metabolic tissue and in a BLCA signaling model 33-35.	B, with partial A support in bladder	Direct measurement of SIRT1 protein and deacetylase activity after PPARγ perturbation in urothelial models
6	SIRT1 directly deacetylates GPX4	GPX4 acetylation has been directly demonstrated in non-urothelial systems, including functionally characterized K90 acetylation and SIRT3-dependent regulation of mitochondrial GPX4 acetylation 39-41. However, GPX4 has not been established as a SIRT1 substrate.	C for the SIRT1-specific claim	Demonstration of SIRT1-GPX4 interaction; site-specific GPX4 acetylation after SIRT1 gain- and loss-of-function; catalytic-dead SIRT1 controls; and rescue with acetylation-site mutants.
7	GPX4 acetylation limits its ubiquitin dependent degradation	GPX4 acetylation is biologically functional, but reported effects are context- and site-dependent 39-41. Acetylation and ubiquitination compete at shared lysines in other substrates 36; GPX4 is subject to ubiquitin dependent regulation 37, 38. The two observations have not been connected.	C	Lysine substitution mutants assessed for ubiquitination, half-life and ferroptosis sensitivity
8	PPARγ suppression sensitizes BLCA cells to platinum through ferroptosis	PPARγ inhibition or degradation reduces proliferation, survival, or motility in urothelial models 14, 15, 44, but ferroptosis was not the mechanistic endpoint in these studies, and a PPARγ-GPX4-ferroptosis mechanism of platinum sensitization has not been directly established.	C	Platinum combined with genetic or pharmacological PPARγ suppression in urothelial cell lines and patient-derived models, with GPX4 expression, lipid-peroxidation, ferroptosis-rescue, and GPX4-rescue experiments.

## Abbreviations

Ac: Acetylation
ACSL4: Acyl-CoA synthetase long-chain family member 4
ADC: Antibody-drug conjugate
AR: Androgen receptor
ATAC-seq: Assay for transposase-accessible chromatin using sequencing
BLCA: Bladder cancer
ChIP-qPCR: Chromatin immunoprecipitation followed by quantitative polymerase chain reaction
ChIP-seq: Chromatin immunoprecipitation sequencing
CoQ: Coenzyme Q
CUT&RUN: Cleavage under targets and release using nuclease
DNA: Deoxyribonucleic acid
DXd: Deruxtecan, a topoisomerase I inhibitor payload
EMP1: Epithelial membrane protein 1
FDA: Food and Drug Administration
FGFR3: Fibroblast growth factor receptor 3
FSP1: Ferroptosis suppressor protein 1
GPX4: Glutathione peroxidase 4
H3K27ac: Histone H3 lysine 27 acetylation
HER2: Human epidermal growth factor receptor 2
IHC: Immunohistochemistry
MIBC: Muscle-invasive bladder cancer
MMAE: Monomethyl auristatin E
mRNA: Messenger RNA
MVAC: Methotrexate, vinblastine, doxorubicin, and cisplatin
MYC: MYC proto-oncogene, bHLH transcription factor
NAD⁺: Nicotinamide adenine dinucleotide, oxidized form
NCOR: Nuclear receptor corepressor
Nrf2: Nuclear factor erythroid 2-related factor 2
PLA2G2F: Phospholipase A2 group IIF
POLD1: DNA polymerase delta catalytic subunit 1
PPARγ: Peroxisome proliferator-activated receptor gamma
PRISMA: Preferred Reporting Items for Systematic Reviews and Meta-Analyses
ROS: Reactive oxygen species
RXR: Retinoid X receptor
SIAH1/2: Siah E3 ubiquitin protein ligase 1/2
SIRT1: Sirtuin 1
SIRT3: Sirtuin 3
SLC7A11: Solute carrier family 7 member 11
SMRT: Silencing mediator for retinoid and thyroid hormone receptors
TCGA: The Cancer Genome Atlas
TM4SF1: Transmembrane 4 L six family member 1
